# Foxd4l1.1 Negatively Regulates Chordin Transcription in Neuroectoderm of *Xenopus* Gastrula

**DOI:** 10.3390/cells10102779

**Published:** 2021-10-17

**Authors:** Vijay Kumar, Ravi Shankar Goutam, Zobia Umair, Soochul Park, Unjoo Lee, Jaebong Kim

**Affiliations:** 1 Department of Biochemistry, Institute of Cell Differentiation and Aging, College of Medicine, Hallym University, Chuncheon 24252, Korea; vijay10187@gmail.com (V.K.); ravi2005gautam@gmail.com (R.S.G.); zobiamughal@gmail.com (Z.U.); 2 Department of Molecular Medicine, School of Medicine, Gachon University, Incheon 21999, Korea; 3 Department of Biological Sciences, Sookmyung Women’s University, Seoul 04310, Korea; scpark@sookmyung.ac.kr; 4 Department of Electrical Engineering, Hallym University, Chuncheon 24252, Korea

**Keywords:** Chrd, Foxd4l1.1, Smad2, Smad3, neural repressor, transcription regulation, *Xenopus*

## Abstract

Inhibition of the bone morphogenetic proteins (BMPs) is the primary step toward neuroectoderm formation in vertebrates. In this process, the Spemann organizer of the dorsal mesoderm plays a decisive role by secreting several extracellular BMP inhibitors such as Chordin (Chrd). Chrd physically interacts with BMP proteins and inhibits BMP signaling, which triggers the expression of neural-specific transcription factors (TFs), including Foxd4l1.1. Thus, Chrd induces in a BMP-inhibited manner and promotes neuroectoderm formation. However, the regulatory feedback mechanism of Foxd4l1.1 on mesodermal genes expression during germ-layer specification has not been fully elucidated. In this study, we investigated the regulatory mechanism of Foxd4l1.1 on *chrd* (a mesodermal gene). We demonstrate that Foxd4l1.1 inhibits *chrd* expression during neuroectoderm formation in two ways: First, Foxd4l1.1 directly binds to FRE (Foxd4l1.1 response elements) within the *chrd* promoter region to inhibit transcription. Second, Foxd4l1.1 physically interacts with Smad2 and Smad3, and this interaction blocks Smad2 and Smad3 binding to activin response elements (AREs) within the *chrd* promoter. Site-directed mutagenesis of FRE within the *chrd(-2250)* promoter completely abolished repressor activity of the Foxd4l1.1. RT-PCR and reporter gene assay results indicate that Foxd4l1.1 strongly inhibits mesoderm- and ectoderm-specific marker genes to maintain neural fate. Altogether, these results suggest that Foxd4l1.1 negatively regulates *chrd* transcription by dual mechanism. Thus, our study demonstrates the existence of precise reciprocal regulation of *chrd* transcription during neuroectoderm and mesoderm germ-layer specification in *Xenopus* embryos.

## 1. Introduction

Gastrulation is the central event of vertebrate embryonic development, allowing the formation of three germ layers. In this process, the Spemann organizer of the dorsal mesoderm plays a crucial role by influencing the neighboring ectodermal (epidermal) cells to achieve neural fate [1,2]. Ectodermal cells express high levels of bone morphogenetic protein 4 (BMP4) that control ectodermal fate by upregulating the expression of several ectodermal specifiers, including *ventx1.1* and *ventx1.2* [3,4]. In turn, the organizer releases potent BMP inhibitors (such as Chrd and Noggin (Nog)) to block BMP signaling in the surrounding embryonic region. The reciprocal repression between the ectoderm (BMP signaling) and the dorsal mesoderm (BMP antagonism) is integrated for controlling neuroectoderm formation and overall embryonic patterning. Several reports have demonstrated that BMP antagonists secrete from the dorsal mesoderm, physically interact with BMP proteins, and block cognate signaling in the adjacent region [5,6]. As shown previously, BMP downstream repressor Ventx1.1 maintains and reinforces the gradient by suppressing the targeted neural and dorsal genes in the ventral region of *Xenopus* embryos [7,8,9,10].

Foxd4l1.1 (also known as Foxd5a/b) is an immediate-early neural marker whose expression begins under the BMP inhibited condition in the prospective neuroectoderm, which is necessary for maintaining neural stemness and neural induction [11,12]. We have shown that Foxd4l1.1 directly inhibits the ectodermal fate by binding to FRE (Foxd4l1.1 response element) within the *ventx1.1* promoter region [13]. Foxd4l1.1 physically interacts with Xbra, and it blocks Xbra interacting with Smad1 to interact with and activate *ventx1.1* transcription. [13]. In addition, Foxd4l1.1 promotes inhibitory phosphorylation at the linker region of Smad1 and reduces Smad1 *C*-terminal activatory phosphorylation [13]. These results point to Foxd4l1.1 inhibiting target gene transcription at multiple levels. For negative regulation, BMP/Ventx1.1 strongly inhibits *foxd4l1.1* expression and neural differentiation in the ectodermal region. The *foxd4l1.1* promoter contains Ventx1.1 response elements (VREs) within its proximal region where Ventx1.1 binds and inhibits transcription [14,15]. These reciprocal inhibitory pathways involving Foxd4l1.1 contribute to the fine-tuning of neural and non-neural fate specification. Similarly, downregulation of BMP signaling by ectopic expression of dominant-negative BMP receptor (DNBR) induces the expression of early neural target genes *foxd4l1.1* and *zic3* [14,15].

Via reciprocal inhibition, these results indicate that BMP/Ventx1.1 and Foxd4l1.1 regulate opposite signaling during gastrulation. Such an observation provides for a potential explanation of how ectodermal genes remain silent in neuro-ectoderm. Previous studies indicate that expression of Chrd from dorsal mesoderm is an essential step toward neural induction and neuroectoderm formation in BMP inhibited conditions [16,17,18]. The primary roles of Chrd in BMP inhibition and neuroectoderm formation have been documented in depth. However, the feedback regulation underlying Foxd4l1.1 mediated regulation of mesodermal gene(s) remains mostly unexplored.

In this study, we hypothesized that the Foxd4l1.1 might inhibit the organizer-specific gene expression during neural specification in order to maintain neural identity. We found that Foxd4l1.1 inhibits dorsal mesoderm specific genes *chrd*, *nog*, and *goosecoid* (*gsc*) expression. Based on Foxd4l1.1 genome-wide ChIP sequencing, we chose *chrd* as a potential target gene to investigate the Foxd4l1.1 regulatory mechanism at the transcriptional level. The results suggest that once Foxd4l1.1 is expressed in naive ectoderm, it can regulate transcription of target genes differentially. Here, we demonstrate that Foxd4l1.1 inhibits *chrd* transcription by a dual mechanism. First, Foxd4l1.1 binds to FRE (Foxd4l1.1 response elements) within the *chrd* promoter and inhibits its transcription. Second, Foxd4l1.1 physically interacts with the *C*-terminal MH2 domain of Smad2 and Smad3 (Smad2/3) and blocks their activator activity on *chrd* transcription. We have previously reported that both Smad2/3 bind to AREs (activin response elements 1 and 2) within the *chrd* promoter to activate transcription [19]. Reporter gene assays and ChIP-PCR results indicate that co-injected Foxd4l1.1 blocks Smad2/3 binding with AREs. Altogether, these results provide evidence of a reciprocal inhibitory mechanism for neuroectoderm versus mesoderm specification. These data point to transcriptional regulation of certain non-neural factors being tightly controlled in a spatiotemporal manner as required for the normal development of vertebrate embryos.

## 2. Materials and Methods

### 2.1. Ethics Statement

The animal studies were conducted in accordance with the Institutional Animal Care and Use Committee (IACUC) regulations of Hallym University (Hallym 2019-79, 2019-80). All our research team members attended educational and training courses for the appropriate care and use of the experimental animals. Adult *X. laevis* were maintained in suitable containers under a 12 h light/dark (LD 12:12 h) cycle at 18 °C, tended by authorized personnel and according to the Institute of Laboratory Animal Guidelines Resources of Hallym University.

### 2.2. Cloning of Chrd Genomic DNA

The *chrd* 2250 bps long promoter region was cloned into a pGL3-basic plasmid (Promega, USA) as described previously [19]. The cloned construct is referred to as *chrd(-2250)luc (*or *chrd(-2250)*).

### 2.3. DNA and RNA Preparation

The Flag-Foxd4l1.1, HA-Smad2 (wild type and truncated), and HA-Smad3 (wild type and truncated) mRNAs used in the study were constructed by linearizing the target vectors using the Acc65I restriction enzyme. The linearized vectors pCS4-Flag-Foxd4l1.1, pCS4-HA-Smad2/3 (wild type and truncated) were used in the in vitro transcription assays and using the MEGA script kit (Ambion, Austin, TX, USA), according to the manufacturer’s instructions. Synthetic mRNAs were quantified at 260/280 nm using a spectrophotometer (SpectraMax, Molecular Devices, San Jose, CA, USA); they were diluted in DEPC water to a final concentration of 1 ng/5 µL and stored at −80 °C for further use.

### 2.4. Promoter Constructs

The *chrd(-2250)* construct was used to design the serially deleted promoter constructs, as shown in Table 1 and Figure 3B. Restriction enzymes NheI (upstream) and XhoI (downstream) (Promega) were used for PCR amplification of the regions of interest, which were then subcloned into the digested pGL3-basic plasmid. For *chrd(-2250)eGFP* construct, the luciferase coding region was replaced by *eGFP* in the pGL3-basic vector using NcoI/XbaI (Promega) restriction enzymes. Similarly, activin response element (ARE) and BMP response element (BRE) were used in this study. Both ARE and BRE had been previously cloned into pGL3-basic vectors, shown to specifically respond to activin and BMP signaling, respectively [20,21].

### 2.5. Embryo Injection and Explants Culture

Adult *X. laevis* were obtained from the Korean *Xenopus* Resource Center for Research. The oocytes were obtained by injecting *X. laevis* females with 500 units of human chorionic gonadotropin hormone (Sigma, St. Louis, MO, USA). As previously described, the obtained oocytes were fertilized in vitro, and microinjection was performed at the one-cell stage into the animal pole of the embryos with the specified DNA or RNA [22]. The injected whole embryos were cultured in 30% Marc’s Modified Ringer’s (MMR) solution and harvested at stage 11 for further experiments.

### 2.6. RT-PCR

Total RNA was isolated from whole embryos using the TRIzol reagent and following the manufacturer’s instructions (Ambion, Austin, TX, USA). Isolated RNA samples were treated with DNase I to remove genomic DNA contamination. Per manufacturer’s instructions, RT-PCR was performed with 1 μg total RNA per reaction using Superscript-IV (Invitrogen, Waltham, MA, USA). Thermal cycling was performed as follows: 30 s at 95 °C, 30 s at each annealing temperature, 30 s at 72 °C, and 20–30 cycles of amplification with the primers, as indicated in Table 2.

### 2.7. Luciferase Assays

The *chrd(-2250)* construct along with the serially deleted and mutant domain constructs were each injected with or without Foxd4l1.1 and Smad2/3 mRNA and reporter assays were then performed as previously described [23]. The relative promoter activities via the reporter assays were measured using a luciferase assay system and according to the manufacturer’s instructions (Promega, Madison, WI, USA). Five different sets of embryos (3 embryos/group) were harvested at stage 11 and homogenized in 10 μL lysis buffer/embryo. Embryo homogenates (10 μL each) were combined with a 40 μL luciferase substrate, and the reporter gene activity was measured using an illuminometer (Berthold Technologies, Bad Wildbad, Germany). All experiments were separately performed at the minimum in triplicate.

### 2.8. Smad2- and Smad3-Truncated Protein Construct

Both Smad2 and Smad3 (Smad2/3) sequences were of human origin and described previously (hSmad2 and hSmad3) [24]. For the Smad2/3-ΔC (*C*-terminal deleted)-truncated protein constructs, the forward primer coded from the start codon (ATG), and the reverse primer coded from the last codon of the linker region of hSmad2/3. For the Smad2/3-C-ter (only *C*-terminal in which *N*-terminal and linker region was deleted) truncated protein, the forward primer was from the first codon of the *C*-terminal domain, and the reverse primer was from the last codon of the *C*-terminal of hSmad2/3. The open reading frame (ORF) for each recombinant hSmad2/3 construct was determined according to the primers used with the primers listed in Table 3, and the map of the recombinant proteins is shown in Figure 4C.

### 2.9. Co-Immunoprecipitation and Western Blotting

Embryos were co-injected with Flag-foxd4l1.1, HA-smad2, and HA-smad3 (or HA-smad2- and HA-smad3-truncated protein) constructs at the one-cell stage. The embryos were harvested at stages 10.5 or 11 and then homogenized in lysis IP buffer as previously described [3]. The cleared cell lysates were incubated overnight at 4 °C with anti-Flag (mouse, F1804, Sigma, St. Louis, MO, USA), anti-HA (rabbit, C29F4, Cell Signaling Technology, Danvers, MA, USA), normal IgG (mouse, L1216, Santa Cruz Biotechnology, Dallas, TX, USA) or normal IgG (rabbit, Cell Signaling Technology). The immunocomplexes were then precipitated by protein A/G beads (SC-2003, Santa Cruz Biotechnology). Western blotting was performed on separated proteins from 10% (wild-type protein constructs) and 14% (truncated HA-smad2 and HA-smad3 constructs) SDS–polyacrylamide gels. The membranes were blocked in 5% skim milk (7262735, BD DIFCO), washed, and incubated with species-specific antibodies as described previously [3,13]. Immunocomplexes were then visualized by using an ECL detection kit (GE Healthcare).

### 2.10. Site-Directed Mutagenesis

The mutations were performed using a site-directed mutagenesis Kit (Muta-Direct, iNtRON Biotechnology, Seoul, Korea) and, as previously described, with specific primer oligonucleotides (shown in Table 4), following the manufacturer’s instructions [23].

### 2.11. Chromatin Immunoprecipitation

ChIP assay was performed as in a previous study [25]. The mRNAs encoding Flag-Foxd4l1.1 and HA-Smad2/3 (1 ng/embryo) were injected at the one-cell stage. The injected embryos were harvested at stage 11 (100 to 125 embryos/sample), and crosslinking was performed in 1.85% formaldehyde solution (Sigma-Aldrich, St. Louis, MO, USA). RIPA buffer containing proteinase inhibitor cocktail (Thermo Fisher, Waltham, MA, USA) was added to fixed embryos, followed by homogenization and sonication for 90 s with 2 short intervals every 30 s to produce 200 to 300 base pair long fragments (Omni Sonic Ruptor 400). The anti-Flag and anti-HA polyclonal antibody (SC-805, Santa Cruz Biotechnology, Dallas, Texas, USA) or normal mouse IgG (SC-2025, Santa Cruz Biotechnology, Dallas, Texas, USA) were used to immunoprecipitate chromatin. The precipitated chromatin was then heated overnight at 65 °C to reverse the crosslinks, and the DNA was purified for further use. The ChIP-PCR was then performed with immunoprecipitated chromatin using region-specific primers (shown in Figure 5A). The primers used are listed in Table 5.

### 2.12. ChIP-Sequencing Analysis

The Foxd4l1.1 mRNA (1 ng/embryo) was injected at the one-cell stage, and embryos (approximately 1000 to 1200 embryos) were harvested at stage 11. ChIP assay was performed accordingly to a previously described method [25] and as stated in Section 2.11. Total immunoprecipitated chromatin was sequenced by (Macrogen, Seoul, South Korea), with raw data (short reads) received in FASTA format. The sequencing data were uploaded to Galaxy server (https://usegalaxy.org, accessed on 14 June 2020) an online tool for further analysis. The FASTA files were groomed by FASTQ Groomer (NGS:Tools), and mapping was performed with the reference genome (*Xenopus laevis* genome version 9.1, xenbase.org) using Bowtie for Illumina (NGS:Mapping) [26,27]. The coverage dataset for chromosome 5s was split from Pileup dataset as described previously [28]. Finally, the Foxd4l1.1 coverage within the *chrd* genomic region (Xla.v9.1, from chr5S:81716234 (−3000 bps) to chr5S:81726234 (+7000 bps)) was plotted, as shown in Figure 3A.

### 2.13. eGFP Fluorescence

The *chrd(-2250)eGFP* construct was injected (200 pg/embryo) with or without Foxd4l1.1 or Smad2 mRNA (1 ng/embryo) at the one-cell stage and into the animal hemisphere. eGFP fluorescence was assessed using a stereo microscope with a royal blue light adapter (Stereo Microscope Fluorescence Adapter, NIGHTSEA, Lexington, MA, USA), and photographs were captured using a Nikon D810 camera (Nikon, Japan).

### 2.14. Statistical Analysis

The obtained data were analyzed using unpaired two-tailed Student’s *t*-test or ordinary one-way ANOVA using GraphPad Prism 9.0 (GraphPad, San Diego, CA, USA). The error bars within the graphs represent mean + standard deviation (SD) of three independent experiments. For fold-change values, the relative light units (RLUs) of three independent samples were calculated. For example, RLUs were divided by average values (control sample); however, the RLU values of treated samples were divided by average values of the control samples (separately). Significance values were set as ** for *p* ≤ 0.01, *** for *p* ≤ 0.001, and **** for *p* ≤ 0.0001; n.s. denotes non-significant values.

## 3. Results

### 3.1. Foxd4l1.1 Inhibits Chrd and Dorsal Target Gene Expression

**Figure 1:**
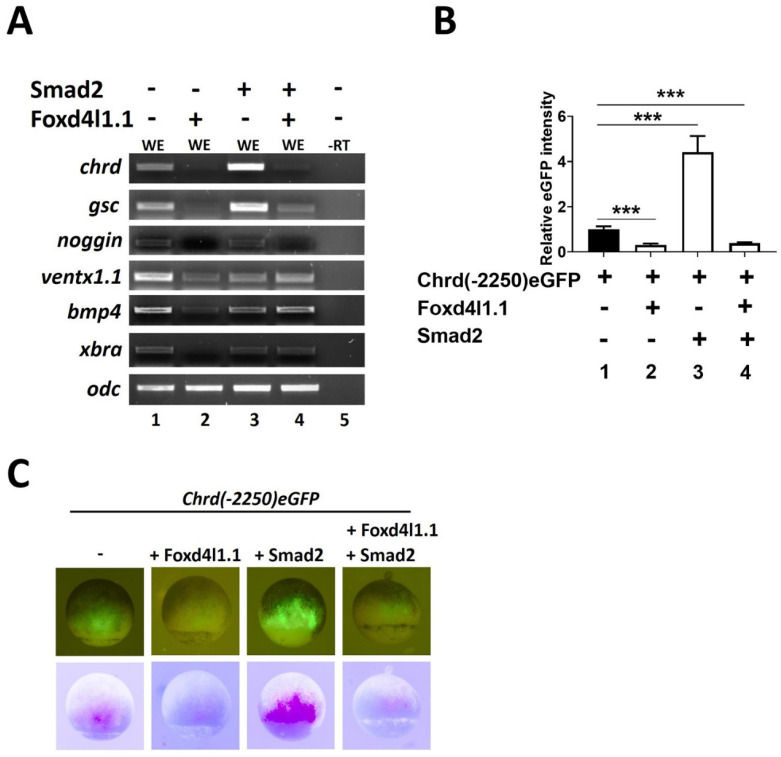
Ectopic expression of Foxd4l1.1reduced dorsal mesoderm (organizer) and ectodermal gene expression during gastrula. *Xenopus* embryos were injected with Foxd4l1.1 and Smad2 (1 ng/embryo) at the one-cell stage. RT expression was analyzed at stage 11 in whole embryos (WE): (**A**) RT expression of different germ-layer-specific markers was analyzed; (**B** and **C**) *chrd(*−*2250)eGFP* was injected alone or co-injected with Foxd4l1.1, Smad2, or both at the one-cell stage; eGFP fluorescent analysis was performed at stage 11: (**B**) quantification of *chrd(*−*2250)eGFP* fluorescent intensity (bar 1) co-injected with Foxd4l1.1 (bar 2), co-injected with Smad2 (bar 3), and Foxd4l1.1 plus Smad2 (bar 4); (**C**) *chrd(*−*2250)eGFP* fluorescence along with co-injection with Foxd4l1.1 or Smad2 or both, separately (at stage 10/10.5) as indicated. In the lower panel, inverted color images generate the pink color for eGFP fluorescent positive areas and reduce the background seen. -RT (No RT) served as a negative control, and uninjected whole embryo (WE) served as a positive control. *** *p* ≤ 0.001.

Foxd4l1.1 ectopic expression induces early neural markers including *sox2*, *sox3*, *sox11*, *geminin*, and *zic2* [11,12] and several late neural-specific genes including *ncam*, *ngnr*, *otx2*, *krox20*, and *hoxb9* [13]. Foxd4l1.1 acts as a repressor for non-neural cells, and inhibits transcription of target genes; for example, Foxd4l1.1 binds within the *ventx1.1* promoter region and inhibits its transcription [13]. In this study, we report ectopic Foxd4l1.1-inhibiting expression of dorsal mesoderm or organizer-specific genes *chrd*, *gsc*, *nog*, and *xbra* (Figure 1A, lane 1 vs. 2). We also examined the ectopic expression of Foxd4l1.1-inhibiting epidermal target genes *bmp4* and *ventx1.1* (Figure 1A, lane 1 vs. 2), as previously reported [13]. Several studies have demonstrated that Smad2 induces mesoderm genes *chrd*, *gsc*, and *nog*, as well as neural *foxd4l1.1*, *zic3*, *ncam* expression in *Xenopus* embryos [13,19,23]. Similarly, Smad2 induces the expression of *chrd, gsc*, and *nog* (Figure 1A, lane 1 vs. 3). However, Smad2 co-injected with Foxd4l.1 was not sufficient to fully rescue the *chrd*, *gsc*, and *nog* expression (Figure 1A, lane 3 vs. 4), suggesting Foxd4l1.1 being an important inhibitor of mesoderm and ectoderm. We observed that *smad2* significantly recovers *xbra* expression when co-injected with Foxd4l1.1 (Figure 1A, lane 3 vs. 4). These results raised the possibility that Foxd4l1.1 may act as a repressor for these genes, as it was previously reported that Foxd4l1.1 represses *ventx1.1* during neuroectoderm formation [13]. We thus examined the effect of Foxd4l1.1 and Smad2 on *chrd(-2250)eGFP* reporter constructs. Foxd4l1.1 injected embryos had markedly reduced *chrd(-2250)eGFP* expression (5 fold) in terms of GFP fluorescence (Figure 1B, bar 1 vs. bar 2 and Figure 1C, the first vs. second panel). As expected, Smad2 injected embryos showed markedly increased *chrd(-2250)eGFP* expression (Figure 1B, bar 1 vs. bar 3 and Figure 1C, the first vs. third panel). However, embryos having Foxd4l1.1 co-injected with Smad2 showed significantly reduced (3.33 fold) *chrd(-2250)eGFP* expression (Figure 1B, bar 1 vs. bar 4 and Figure 1C, the first vs. fourth panel). Together, these results indicate that Foxd4l1.1 strongly inhibits the *chrd* endogenous and reporter gene (*chrd(-2250)eGFP)* expression in *Xenopus* embryos.

### 3.2. Foxd4l1.1 Abolishes Mesoderm and Ectoderm Specific Reporter Genes Expression

**Figure 2:**
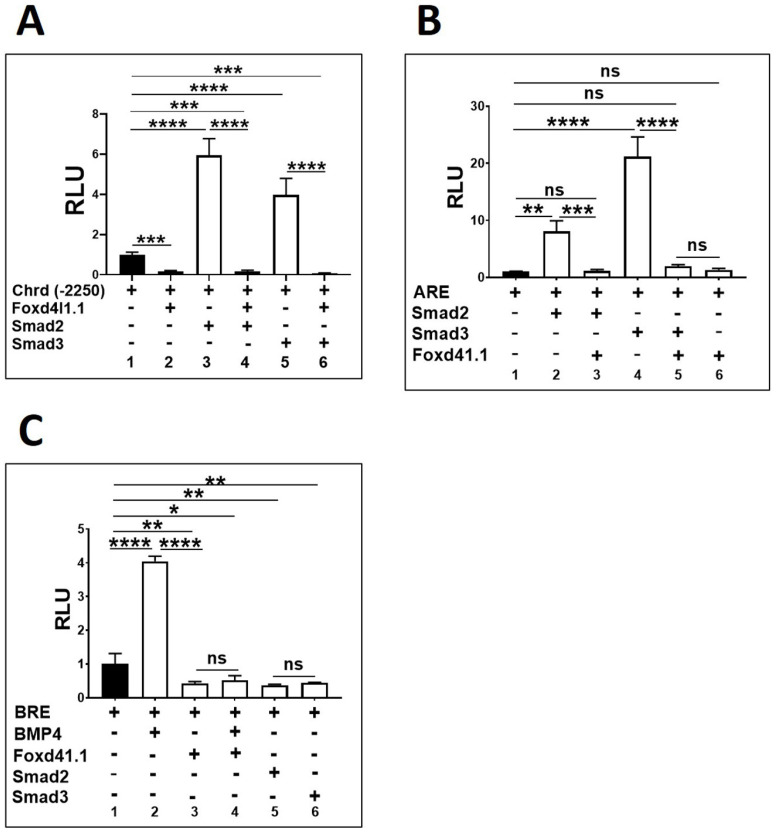
Ectopic presence of Foxd4l1.1 abolished *chrd(*−*2250)*, *ARE,* and *BRE* mediated expression. *Xenopus* embryos were injected with *chrd(*−*2250)*, ARE, and BRE (40 pg/embryo) with or without Foxd4l1.1, Smad2/3, or BMP4 mRNA (1 ng/embryo) at the one-cell stage and harvested at stage 11: (**A**) *chrd(*−*2250)* reporter activities with or without Foxd4l1.1 or Smad2/3; (**B**) ARE reporter activities with or without Foxd4l1.1 or Smad2/3; (**C**) BRE reporter activities with or without BMP4, Foxd4l1.1, or Smad2/3. The y-axis represents relative light units (RLUs). * *p* ≤ 0.05, ** *p* ≤ 0.01, *** *p* ≤ 0.001, and **** *p* ≤ 0.0001, n.s. denotes non-significant values.

Smad2 and Smad3 activate chrd transcription via direct binding to AREs within the *chrd* promoter region [19]. We further examined any Foxd4l1.1 effects on *chrd(-2250)* reporter activity. We found that *chrd(-2250)* expression is significantly reduced ( 5 fold) by the added expression of Foxd4l1.1 (Figure 2A, bar 1 vs. 2). These results are similar to those we observed previously with *chrd(-2250)eGFP* (Figure 1). We next tested the Foxd4l1.1 effect on reporter activity when combined with Smad2/3. Smad2 or Smad3 alone significantly induces reporter activity (6 fold and 4 fold, respectively) (Figure 2A, bar 1 vs. bars 3 and 5). When co-injected with Foxd4l1.1 expressing construct, the Smad2/3 activation of *chrd(-2250)* is abolished (Figure 2A, bars 1 to 6). We needed to confirm whether the Foxd4l1.1 inhibitory effect was unique to chrd or whether it could inhibit other Smad2/3 target genes as well. We used the ARE and BRE described in Section 2.4 to address this question. ARE has been reported to respond to Smad2/3 expression [21]. Similarly, the BRE reporter responded to BMP4 induction [20]. As expected, ectopic expression of Smad2 or Smad3 increases the ARE reporter activity (9 and 20 fold, respectively) (Figure 2B, bar 1 vs. bars 2 and 4). While co-injected with Foxd4l1.1, the Smad2/3 mediated activation of ARE is blocked (Figure 2B, bar 2 vs. bar 3 and bar 4 vs. bar 5). Interestingly, Foxd4l1.1 alone does not affect ARE expression (Figure 2B, bar 1 vs. bar 6), presumably because ARE itself does not contain any FRE. We observed similar effects with Foxd4l1.1 expression on BRE reporter activity in response to BMP4, and the reporter assay indicated that BRE positively responds to BMP4 presence (4 fold) (Figure 2C, bar 1 vs. bar 2), while Foxd4l1.1 co-injected blocks the BMP4-mediated BRE expression (2 fold) (Figure 2C, bar 1 vs. bars 3 and 4). Smad2 and Smad3 also significantly inhibit BRE (2.5 fold) expression (Figure 2C, bar 1 vs. bars 5 and 6).

### 3.3. Chrd Promoter Contains Foxd4l1.1 Response Element (FRE)

**Figure 3:**
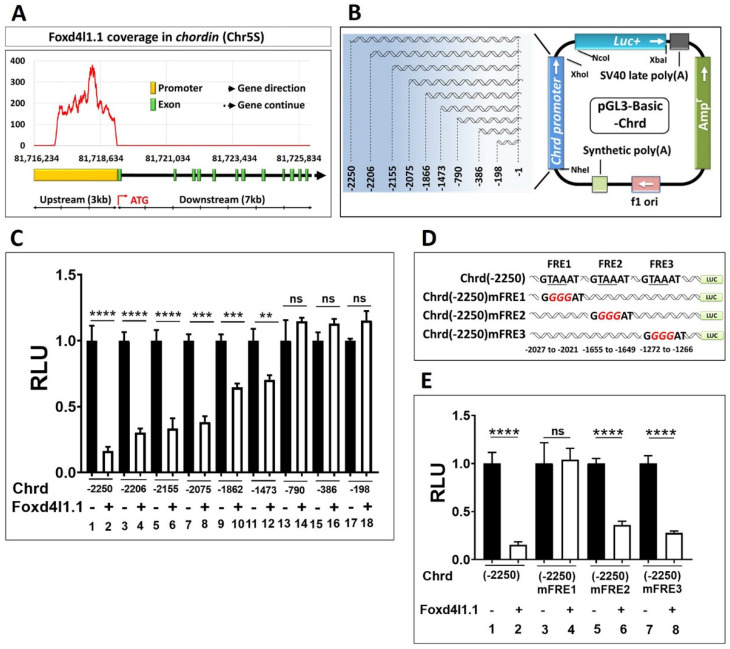
Chrd promoter contains FRE1: (**A**) ChIP-sequencing coverage plot of Foxd4l1.1 within the chrd promoter region; (**B**) schematic representation of the serially deleted promoter constructs of chrd promoter. The numbering of promoter constructs (or *cis*-acting response elements) is given from the translational start site (TLS); (**C**) relative promoter activities of serially deleted promoter constructs of chrd(-2250) with or without Foxd4l1.1; (**D**) systemic representation of mutated FRE1, FRE2, and ARE2 (targeted sequences; underlined, italic, red color) within the region of −2250 bps chrd promoter construct; (**E**) relative luciferase activity for chrd(-2250), chrd(-2250)mFRE1, chrd(-2250)mFRE2, and chrd(-2250)mFRE3 with or without Foxd4l1.1. ** *p* ≤ 0.01, *** *p* ≤ 0.001, and **** *p* ≤ 0.0001, n.s. denotes non-significant values.

We performed ChIP sequencing to identify the Foxd4l1.1 genome-wide targets during Xenopus gastrulation. The obtained data indicated that Foxd4l1.1 targets major mesoderm-specific genes, including *chrd*, *gsc*, *nog2*, similar to ectodermal *ventx1.1* and *ventx1.2*. In this study, we selected *chrd* as the Foxd4l1.1 target for further investigation, and other target genes were not analyzed (data not shown). We plotted the Foxd4l1.1 coverage within a 10 kbs long genomic region of the *chrd* gene (Figure 3A). The coverage plot suggested that the *chrd* promoter may contain functionally active FRE(s) from −2252 bps to +3 bps (Figure 3A). To identify the active FRE(s) within the *chrd(−2250)* promoter, we constructed several serially deleted chrd reporter constructs (Figure 3B) and assayed the reporter activity of these serially deleted promoters with or without Foxd4l1.1 mRNA. As shown, Foxd4l1.1 strongly inhibits the *chrd(−2250)* (6.25 fold), *chrd(−2206)* (4.34 fold), *chrd(−2155)* (3.33 fold), *chrd(−2075)* (2.63 fold), *chrd(−1862)* (1.56 fold), and *chrd(−1473)* (1.42 fold) activity (Figure 3C, bars 1 to 12). However, the promoter constructs smaller than *chrd(-1473)* bps showed no significant changes (Figure 3C, bar 13 to 18). As previously reported, Foxd4l1.1 can act by *cis*-motif binding (RYAAAYA; the conserved sites for forkhead transcription factors across vertebrates) [29,30]. We observed three putative FREs within the chrd promoter, referred to as FRE1 (−2027 to −2021), FRE2 (−1655 to −1649), and FRE3 (−1272 to −1266), as shown in Figure 3D. To identify any functionally active FRE(s), site-directed mutagenesis was performed on the putative sites. Three conserved nucleotides were altered in each FRE candidate (FRE1-3) (GTAAAT to GGGGAT) within the full-length promoter construct *(chrd(−2250)*) (Figure 3D). Effects of these point mutations in the FRE candidates (chrd(−2250)mFRE1, *chrd(−2250)mFRE2*, and *chrd(−2250)mFRE3)* were then examined with or without ectopic Foxd4l1.1. We report that *chrd(−2250)mFRE1* shows no changes with ectopic Foxd4l1.1, while chrd(−2250)mFRE2 and *chrd(−2250)mFRE3* still displays significantly reduced changes (2.77 and 3.70 fold, respectively) when co-injected with Foxd4l1.1 (Figure 3E, bars 1 to 8). Notably, deleted constructs *chrd(−1862)* and *chrd(−1473)* without FRE show a moderate reduction in reporter gene expression (Figure 3C, bars 9 to 12). Similarly, we also observed that mutation of FRE2 and FRE3 show slightly less reduction in reporter assay by ectopic Foxd4l1.1. The possible explanation of this observation could be that since FRE2 and FRE3 are located between −1862 and −790 bps they may act as response elements for another potential repressor of the forkhead family. Collectively, these results suggest that FRE1 is a functionally active *cis*-acting element in which Foxd4l1.1 binds and inhibits transcription activation in the chrd promoter.

### 3.4. Foxd4l1.1 Physically Interacts with Smad2/3 via C-Ternimal MH2 Domain

**Figure 4:**
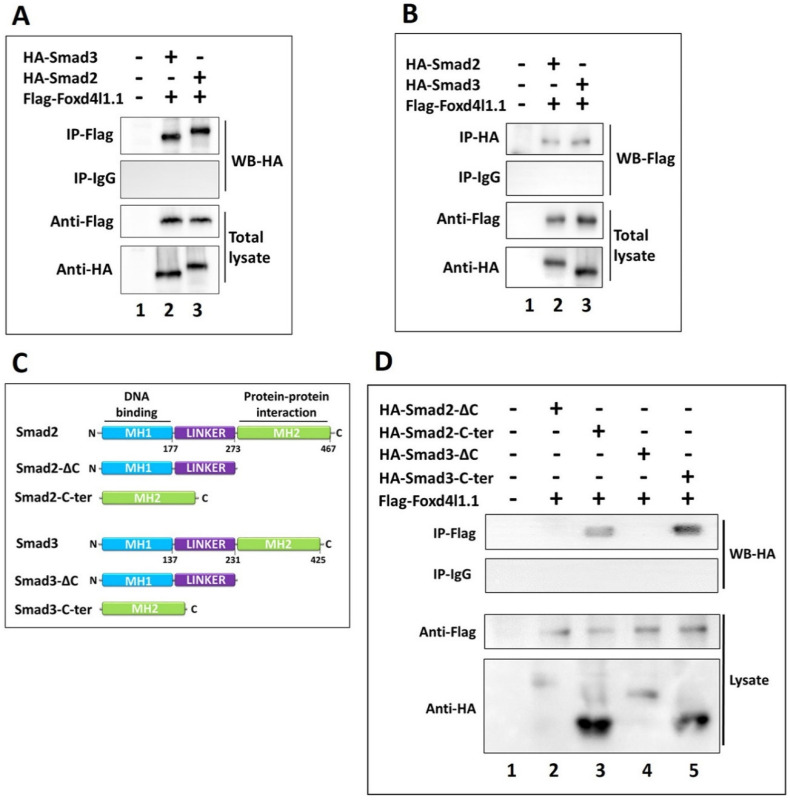
Foxd4l1.1 physically interacts with *C*-terminal domain of Smad2 and Smad3 proteins: (**A**,**B**) co-immunoprecipitation (Co-IP) assay was performed to examine the interaction of Flag-Foxd4l1.1 and HA-Smad2/3 recombinant proteins: (**A**) Co-IP was performed with an anti-Flag antibody, followed by Western blotting (WB) with anti-HA antibody; (**B**) Co-IP was performed with anti-HA antibody, followed by WB with anti-Flag antibody; (**C**) schematic representation of truncated Smad2 and Smad3 constructs; these include Smad2/3-ΔC, having the *C*-terminal domain deleted, and Smad2/3-C-ter, containing only the *C*-terminal domain of the original parental protein; (**D**) co-immunoprecipitation (Co-IP) assay was performed to examine the interaction between Flag-Foxd4l1.1 and HA-Smad2-ΔC, HA-Smad3-ΔC, HA-Smad2-C-ter, and HA-Smad3-C-ter. Co-IP was performed with anti-Flag antibody, followed by WB with anti-HA antibody.

Foxd4l1.1 can inhibit Smad2/3 mediated increases in expression of *eGFP* and similarly a luciferase reporter driven by the *chrd* promoter region ( Figure 1 and Figure 2). A previous study has reported that Foxd4l1.1 interacts with Xbra and blocks Xbra binding to the *ventx1.1* promoter region, inhibiting transcription [13]. We hypothesized that Foxd4l1.1 may also physically interact with Smad2/3 and block binding on AREs within the *chrd* promoter. To test this hypothesis, Flag-Foxd4l1.1, HA-Smad2, and HA-Smad3 mRNA were co-injected in one-cell stage embryos. Co-immunoprecipitation (Co-IP) assays were performed at stage 11 with anti-Flag and anti-HA antibodies. We performed Co-IP in two ways: First, we immunoprecipitated with anti-Flag antibody for Western blotting with anti-HA antibody to indicate any interaction of Foxd4l1.1 with Smad2 and Smad3 (Figure 4A, lane 2 and 3). Second, we immunoprecipitated with anti-HA antibody and performed Western blot with anti-Flag antibody. Both ways yield similar results, demonstrating the interaction of Foxd4l1.1 with Smad2 and Smad3 (Figure 4B, lanes 2 and 3). It has been known that both Smad2 and Smad3 contain a *C*-terminal domain, known as MH2, responsible for protein–protein interactions [31]. To determine the Foxd4l1.1-interacting domain in the two Smads, we designed truncated Smad2/3 protein constructs (separately). These were Smad2/3-ΔC (*C*-terminal deleted) truncated and Smad2/3-C-ter (*N*-terminal and linker region deleted) constructs (shown in Figure 4C). We performed Co-IP to check whether Foxd4l1.1 interacts with Smad2/3-ΔC or Smad2/3-C-ter. The results suggest that Foxd4l1.1 interacts only with the *C*-terminal portion of Smad2/3 (Smad2-C-ter and Smad3-C-ter) (Figure 4D, lanes 3 to 5); meanwhile, truncated protein constructs without having *C*-terminal Smad2/3 (Smad2/3-ΔC) show no interaction with wild-type Foxd4l1.1 (Figure 4D, lanes 2 to 4). Based on these results, we conclude that Foxd4l1.1 directly interacts with the *C*-terminal MH2 domain of Smad2 and Smad3.

### 3.5. Foxd4l1.1 Binds to FRE1 and Blocks Smad2/3 Binding to AREs (ARE1 and ARE2) within the Chrd Promoter

**Figure 5:**
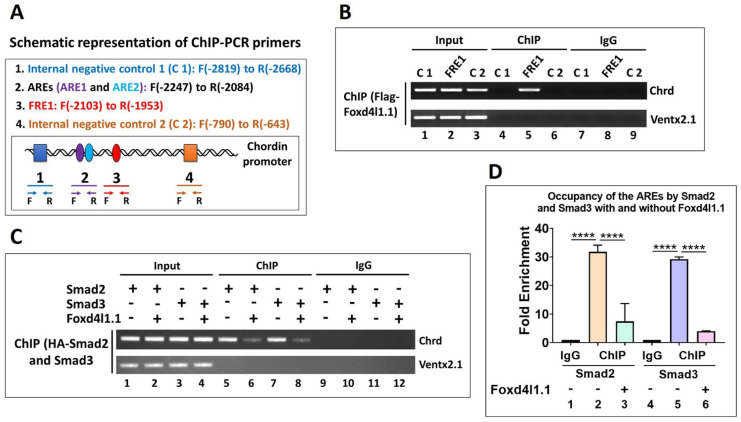
Foxd4l1.1 is bound to FRE1 and blocks Smad2/3 binding to AREs (ARE1 and ARE2). For ChIP-PCR, Flag-Foxd4l1.1 with or without HA-Smad2 and HA-Smad3 mRNA were injected at the one-cell stage, then harvested at stage 11: (**A**) ChIP-PCR primer design and C 1, ARE1, ARE2, FRE1, and C 2 locations within the *chrd* promoter are shown with F as location of the forward primer and R as location of the reverse primer; (**B**) chromatin immunoprecipitation assay was performed to examine the interaction of Flag-Foxd4l1.1 with FRE1, the two internal negative controls (C 1 and C 2), and one external negative control (Ventx2.1); (**C**) chromatin immunoprecipitation assay was performed to examine the occupancy of either Smad2 or Smad3 with or without Foxd4l1.1. Ventx2.1 served as external negative control; (**D**), ChIP-qPCR was performed to examine the occupancy of HA-*Smad2* and HA-*Smad3* to AREs with or without Flag-*Foxd4l1.1* (fold enrichment method used to normalize the ChIP-qPCR reads). **** *p* ≤ 0.0001.

Previous results suggested that Foxd4l1.1 inhibits *chrd* gene expression and blocks Smad2/3 mediated transcription activation. Furthermore, ChIP-sequencing data and site-directed mutagenesis revealed that Foxd4l1.1 might bind to FRE1 to block *chrd* transcription. We performed chromatin immunoprecipitation (ChIP) PCR to examine whether Foxd4l1.1 interacts with endogenous *chrd* promoter. Flag-Foxd4l1.1 was injected at the one-cell stage, and total chromatin was precipitated with anti-Flag antibody, followed by PCR (Figure 5B). We observed that the PCR amplicon encompassing the FRE1 region is highly enriched (Figure 5B, lane 5). These results suggest that FRE1 acts as a functionally active *cis*-acting element for Foxd4l1.1. We next asked whether Foxd4l1.1 blocks Smad2/3 binding to AREs within the *chrd* promoter. To address this question, HA-Smad2 and HA-Smad3 constructs were injected with or without the Flag-Foxd4l1.1 construct at the one-cell stage. We then performed ChIP-PCR at stage 11, and total chromatin was subjected to immunoprecipitation (IP) by anti-HA antibody. Precipitated chromatin was quantified by NanoDrop and diluted to equalize for DNA concentration for every sample, including the input and IP samples (as indicated in Figure 5C). Results demonstrated that Smad2 or Smad3 binding to AREs is dramatically reduced by Foxd4l1.1 presence (Figure 5C lane 5 vs. lane 6 and lane 7 vs. lane 8). Similarly, we performed ChIP-qPCR to examine the effect of Foxd4l1.1 on AREs by Smad2 and Smad3. As expected, ectopic Foxd4l1.1 reduced Smad2 (from 31.7 fold to 7.3 fold) and Smad3 (from 29.2 fold to 4.02 fold) binding affinity to AREs (Figure 5D, bars 1 to 6). These results indicate that Foxd4l1.1 interacts with Smad2/3 and blocks their binding to AREs. Overall, these results indicate a dual inhibitory role for Foxd4l1.1 toward regulating *chrd* transcription in *Xenopus* embryos.

## 4. Discussion

This study was designed to address a potential gene regulatory network (GRN) existing between neural and non-neural fate specifications. We focused on investigating the repressive transcriptional activity of Foxd4l1.1 and how Foxd4l1.1 regulates non-neural gene transcription during gastrulation in *Xenopus* embryos. Foxd4l1.1 is highly conserved across species and is the earliest transcription factor (TF) produced in the naive neuroectoderm, in which it plays an essential role in neural differentiation [11,13,29,30,32]. Here, we report that Foxd4l1.1 represses *chrd* transcription during neural tissue formation. Once Foxd4l1.1 is produced, it drives neural specification and inhibits the non-neural factors within the neural territory to maintain neural identity [13]. A pathway of mutual activation and repression between mesodermal and neural factors, necessary for defining normal germ-layer differentiation and patterning, is implied in this work. Below is the discussion on the potential regulatory mechanism of Foxd4l1.1 for mesoderm-specific gene expression from the point of view of *chrd* transcription.

The alleged roles of the extracellular factor Chrd in overall axis patterning and neural-ectoderm formation have been extensively studied [6,33,34,35,36], and the inductive role of dorsal mesoderm (organizer) signaling in vertebrate development has been previously reported [2,17,19,37,38]. Accumulated evidence suggests that Chrd mediated BMP inhibition is essential for neural induction and anterior–posterior patterning in vertebrate development [39,40,41]. Once neural progenitor cells develop from the ectoderm, Foxd4l1.1 acts as the primary neural specifier and maintains the undifferentiated neural fate [11,13]. We have previously reported that Foxd4l1.1 actively inhibits ectodermal-specific BMP/Smad1 mediated activation of *ventx1.1* [13]. This repressive regulation indicates a particular defensive mechanism opposing a non-neural fate inducer. For other fate-inducing factors involved in opposite signaling as part of germ layer specification GRNs, they still remain to be fully elucidated.

In this study, we focused on the properties of the neural repressor Foxd4l1.1 to examine the regulatory function of mesodermal *chrd*. The reasons behind *chrd* selection in this setting are that (1) Chrd is known for its role in neuroectoderm formation, and it is secreted from the dorsal mesoderm that induces neural ectoderm formation in a BMP inhibited manner; (2) analysis of genome-wide targets of Foxd4l1.1 by ChIP-sequencing indicate that Foxd4l1.1 occupies the *chrd* promoter region; (3) ectopic expression of Foxd4l1.1 markedly reduces endogenous *chrd* expression and also reduces the activity of reporter gene expression under the *chrd* promoter; (4) We had previously shown that Foxd4l1.1 abolishes expression of ectoderm-specific TF *ventx1.1* to protect the neural fate. Chrd expression is limited only to the dorsal mesoderm and involuting or migrating cells during early gastrula [19,42], and we previously reported that several TFs, such as Smad2, Smad3, and Ventx1.1, differentially regulate *chrd* transcription in gastrula stage embryos [8,19,23]. As to why *chrd* active transcription remains only in mesodermal cells, the question had not been fully answered. Based on these observations, we hypothesized that Foxd4l1.1 might be a transcription from neural progenitor cells that blocks *chrd* transcription within the neural territory.

Here, we demonstrate that ectopic Foxd4l1.1 inhibits endogenous expression of the organizer genes (*chrd*, *gsc*, and *nog*) plus ventral/ectoderm (*ventx1.1* and *bmp4*), and pan-mesodermal marker *xbra* genes (Figure 1A). The *chrd(-2250)eGFP* expression is significantly reduced by Foxd4l1.1 (Figure 1B,C), suggesting the neural Foxd4l1.1 significantly represses opposite fate target genes (mesoderm and ectoderm genes). In addition, Foxd4l1.1 strongly inhibits reporter genes expression of *chrd(-2250)*, *BRE*, and *ARE* (Figure 2). Foxd4l1.1 is also able to block activation of reporter genes even when they are co-injected with a known activator (Smad2/3) (Figure 2A–C) [19]. These indicate that Foxd4l1.1 strongly inhibits the opposite germ-layer target genes but also promotes the neural-specific genes [13]. Several studies have also reported that during embryonic germ layer specification, specific TF(s) reciprocally inhibit the other factors [13,43,44,45]. Such findings indicate a reciprocal inhibition between different germ layers and germ-layer-specific factors, which is required for normal embryonic development.

We next asked whether Foxd4l1.1 mediated inhibition of *chrd* is direct, or that it occurs indirectly. We showed that Foxd4l1.1 directly interacts with FRE1 located in −2027 to −2021 bps of the *chrd* promoter region (Figure 3). In other pathways, Foxd4l1.1 interacts with Smad2 and Smad3 directly (Figure 4) and blocks their interaction with AREs within the *chrd* promoter (Figure 5). To examine which domain in Smad2/3 is interacting with Foxd4l1.1, we designed and tested truncated versions of Smad2/3 (Figure 4C). The results suggest that the MH2 domain of Smad2/3 interacts with Foxd4l1.1 (Figure 4D). These findings are similar to previous studies, where MH2 domains proved to be functionally responsible for protein–protein interactions for the Smads [46,47]. Some studies have pointed to Foxd4l1.1 reducing the *C*-terminal phosphorylation of Smad1, involved in activation of Smad1 and resulting in downregulation of BMP/Smad1 target genes [11,13]. We observed that Foxd4l1.1 co-injected with Smad2/3 markedly decreases Smad2/3 binding to cis-acting AREs within the *chrd* promoter (Figure 5C,D). Currently, it remains unclear how Foxd4l1.1 changes the Smad2/3 DNA-binding affinity. There could be a number of mechanisms for Foxd4l1.1 action: First, Foxd4l1.1 could reduce the activation state of Smad2/3 by causing de-phosphorylation of the Smad *C*-terminal region or increasing the Smad linker region phosphorylation (inhibitory phosphorylation) similar to previous reports [11,13]. Second, the complex Foxd4l1.1 with Smad2/3 may result in a conformational change to the Smad2/3 DNA-binding domain (*N*-terminal domain), which reduces its DNA-binding affinity to AREs. Third, ectopic Foxd4l1.1 may induce cytoplasmic retention of Smad2/3, therefore reducing the levels of nuclear Smad2/3 [11,13]. A future investigation is required to differentiate between these possibilities. As we focused on *chrd* transcription regulation by Foxd4l1.1 during gastrula stage embryos, Foxd4l1.1 inhibited *chrd(-2250)luc* and *chrd(-2250)eGFP* activities (by 6.25 fold and 3.33 fold, respectively) (Figure 1; Figure 2). These may be due to *chrd(-2250)* containing a putative FRE (−2027 to −2021 bps) as point mutations by site-directed mutagenesis (G*TAA*AT to G*GGG*AT) reverted the repressor activity (Figure 4E).

**Figure 6:**
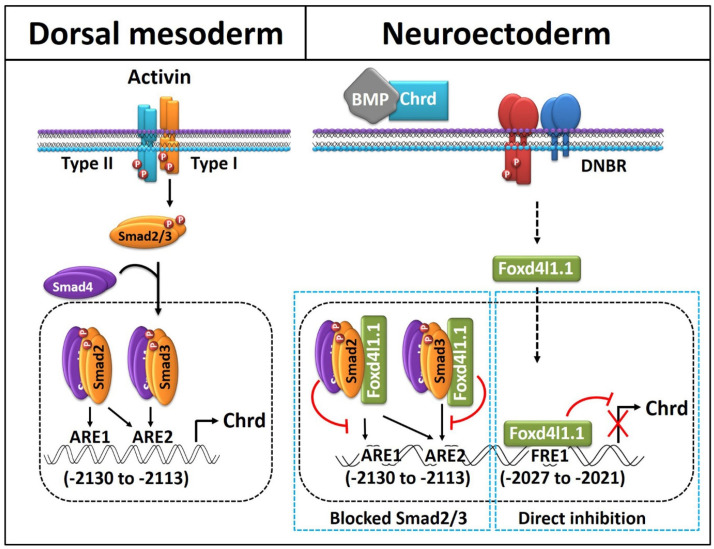
Proposed model of Foxd4l1.1 mediated inhibition of *chrd* transcription. In dorsal mesoderm for the organizer, activin signaling activates *chrd* transcription mediated by Smad2 and Smad3. Each Smad interacts with ARE1 and ARE2 within the *chrd* promoter (−2130 to −2113 bps). The neuroectoderm-specific transcription factor Foxd4l1.1 is directly bound to the FRE1 region of the *chrd* promoter to inhibit *chrd* transcription (−2027 to −2021 bps). Foxd4l1.1 physically interacts with Smad2 and Smad3 and blocks binding to the AREs.

In summary, we demonstrated that neural TF Foxd4l1.1 represses *chrd* transcription in a dual fashion. First is by direct binding to FRE within the 5′ flanking region of *chrd (*−2027 to −2021 bps) promoter. Second, Foxd4l1.1 reduces the DNA-binding affinity of Smad2/3 to AREs. These actions on *chrd* transcription may allow proper neurogenesis or neural tissue formation during development in *Xenopus* embryos. Our results shed light on certain crucial insights into how Foxd4l1.1 regulates certain non-neural factors during neural specification by inhibitory regulation of transcription required for neural versus non-neural tissue formation. With previous findings on inhibitory roles of Foxd4l1.1 toward BRE and ARE, we observed a precise reciprocal inhibition being possible for ectoderm, mesoderm, and neuroectoderm areas. Here, we proposed a scenario of mutual repressive centers acting for completion of different embryonic regions/layers in early gastrula stage embryos in *Xenopus* (Figure 6). We anticipate our study to provide a better understanding of the tightly regulated mechanism of neural fate acquisition in vertebrate embryos.

## Figures and Tables

**Table 1:** Primers used for serially deleted *chrd(-2250)* promoter constructs.

Constructs	Primer Name	Sequence (5′ to 3′)
Upstream primer	Chrd (-2250)_F	GGGGCTAGCGAACGATACTTCAAGGACAAT
	Chrd (-2206)_F	GGGGCTAGCCCACTATCCCCACTAAGATGA
	Chrd (-2155)_F	GGGGCTAGCAGGCATACTTTGGTTTGTGTGT
	Chrd (-2075)_F	GGGGCTAGCTGCAAGTCGAGATCATTGTGT
	Chrd (-1862)_F	GGGGCTAGCAAGAACACAGTGCCAGGCACT
	Chrd (-1473)_F	GGGGCTAGCCAGTAGGTTAGATGAACTACT
	Chrd (-790)_F	GGGGCTAGCACACTCTCTACCCCAATTCT
	Chrd (-386)_F	GGGGCTAGCCTTGACGGCTTTGTTTGCTT
	Chrd (-198)_F	GGGGCTAGCGTGTGGGTACAGAGCAACAA
Downstream primer	Chrd (-2250)_R	GGGCTCGAGTTTTGTGGTTCCAAACGTTCT

**Table 2:** Primers used for RT-PCR amplification of several set of genes.

Gene	Primer Name	Sequence (5′ to 3′)	Cycles
Chrd	Chrd_F	TTAGAGAGGAGAGCAACTCGGGCAAT	25
	Chrd_R	GTGCTCCTGTTGCGAAACTCTACAGA	
Noggin	Noggin_F	AGTTGCAGATGTGGCTCT	27
	Noggin_R	AGTCCAAGAGTCTGAGCA	
Gsc	Gsc_F	GCTGATTCCACCAGTGCCTCACCAG	30
	Gsc_R	GGTCCTGTGCCTCCTCCTCCTCCTG	
Xbra	Xbra_F	GGATCGTTATCACCTCTG	25
	Xbra_R	GTGTAGTCTGTAGCAGCA	
Ventx1.1	Ventx1.1_F	CCTTCAGCATGGTTCAACAG	28
	Ventx1.1_R	CATCCTTCTTCCTTGGCATCTCCT	
Bmp4	BMP4_F	GCATGTACGGATAAGTCGATC	25
	BMP4_R	GATCTCAGACTCAACGGCAC	
ODC	ODC_F	GTCAATGATGGAGTGTATGGATC	25
	ODC_R	TCCATTCCGCTCTCCTGAGCAC	

**Table 3:** Primers used for truncated hSmad2 and hSmad3 protein constructs.

Construct	Primer Name	Sequence (5′ to 3′)	Cycles
Smad2-ΔC	hSmad2_(*N*-terminal)-F	CCCGAATTCCATGTCGTCCATCTTGCCATT	25
	hSmad2_(Linker)-R	GGGCTCGAGTCAAAATGCAGGTTCTGAGT	
Smad2-C-ter	hSmad2_(*C*-terminal)-F	GGTCTCGAGTTATGACATGCTTGAGCAACGC	25
	hSmad2_(*C*-terminal)-R	GGTCTCGAGTTATGACATGCTTGAGCAACGC	
Smad3-ΔC	hSmad3_(*N*-terminal)-F	CCCGAATTCCATGTCGTCCATCCTGCCTTTC	25
	hSmad3_(Linker)-R	AAGTCTAGACCGGCTCGCAGTAGGTAAC	
Smad3-C-ter	hSmad3_(*C*-terminal)-F	CTTGAATTCCTACTCAGAACCTGCATTTTGG	25
	hSmad3_(*C*-terminal)-R	CCATCTAGACTAAGACACACTGGAACAGC	

**Table 4:** Primers used for site-directed mutagenesis.

Mutated Site	Primer Name	Sequence (5′ to 3′)	Cycles
FRE1	*Chrd(*−*2250)mFRE1_F*	TTAAACAGTATAAGGGGATGCTAAAAACACAG	20
	*Chrd(*−*2250)mFRE1_R*	CTGTGTTTTTAGCATCCCCTTATACTGTTTAA	
FRE2	*Chrd(*−*2250)mFRE2_F*	GTCCTGGCATATGAGGGGATTCAGAGCTATCCT	20
	*Chrd(*−*2250)mFRE2_R*	AGGATAGCTCTGAATCCCCTCATATGCCAGGAC	
FRE3	*Chrd(*−*2250)mFRE3_F*	TTCAATCCTTAGCAGGGAATTCCCTCATCTTTC	20
	*Chrd(*−*2250)mFRE3_R*	GAAAGATGAGGGAATTCCCTGCTAAGGATTGAA	

**Table 5:** Primers used for ChIP-PCR amplification.

	Primer Name	Sequence (5′ to 3′)	Cycles
Chrd (Foxd4l1.1_ChIP)	Chrd(FRE)_F	GTTGCTTCTGTTTTCCACCT	25
	Chrd(FRE)_R	GTCTGGCATATCTAGCAGGTC	
Chrd (Internal control 1)	Chrd(C1)_F	TGCGCCGACTAAGTTTCCT	25(qPCR 40)
	Chrd(C1)_R	ATTAGTGACCCATGGCAGG	
Chrd (Internal control 2)	Chrd(C2)_F	ACACTCTCTACCCCAATTCT	25(qPCR 40)
	Chrd(C2)_R	CAGAATGGCATGTGGGAAGA	
Chrd (Smad2 ChIP)	Chrd(AREs)_F	CGATACTTCAAGGACAATTG	24
	Chrd(AREs)_R	AGGTGGAAAACAGAAGCAAC	
Chrd (Smad3 ChIP)	Chrd(AREs)_F	CGATACTTCAAGGACAATTG	24
	Chrd(AREs)_R	AGGTGGAAAACAGAAGCAAC	
Ventx2.1 (External control)	Ventx2.1_F	CTACAGCACTAGCACTGACT	28
	Ventx2.1_R	AGAAAGCTGGAGTTTGGCTGC	

## Data Availability

Original data are available on reasonable request from the corresponding authors.
